# Estimating real-world treatment effects in the presence of measurement error and sparse outcome data using propensity score methods

**DOI:** 10.3389/fphar.2026.1380586

**Published:** 2026-03-09

**Authors:** Jane Burnell, Amitava Banerjee, Gordon Prescott, Chris Sutton, Svetlana Tishkovskaya

**Affiliations:** 1 Lancashire Clinical Trials Unit, Applied Health Research Hub, University of Lancashire, Preston, United Kingdom; 2 Institute of Health Informatics, University College London, London, United Kingdom

**Keywords:** electronic health records, measurement error, oral anti-coagulant, propensity score methods, real-world treatment effect, sparse outcome data, stroke

## Abstract

**Introduction:**

The real-world treatment effect of a novel treatment can be estimated by analysing routinely collected patient data, in the form of Electronic Health Records (EHR). Any treatment allocation in EHR is not randomised and there may be systematic differences between the treatment groups. Propensity Score (PS) methods are commonly used to correct for these differences and reduce the bias in the treatment effect estimate. The aims of the study were to compare the performance of the most popular PS methods in the estimation of the treatment effect in the presence of two common issues in EHRs: covariate measurement error and sparse data.

**Methods:**

The motivational example for this study was the assessment of the treatment effect of the novel oral anti-coagulant Rivaroxaban compared with the previous standard treatment Warfarin for the prevention of future stroke in patients with atrial fibrillation. Using simulation experiments based on a dataset comparing Rivaroxaban with Warfarin, we evaluated the performance of four PS methods.

**Results:**

In the simulations with characteristics of the original dataset, using 3:1 PS matching generated a largest bias of +0.0428 (corresponding ratio of HRs (rHR) 1.0437), whereas for the other PS methods it was smaller and in negative direction: IPTW for ATE -0.0181 (rHR = 0.9821); IPTW for ATT -0.0110 (rHR = 0.9891); PS stratification −0.0099 (rHR = 0.9901), with relative differences between rHRs being small to negligible. Fifty percent under-recording of a covariate (stroke) in the PS model, increased the MSE between 6% and 11% compared to the MSE with no introduced measurement error. While 50% over-recording reduced the MSE by around 35%. The difference in the bias of the low prevalence outcome (0.5%) and the high prevalence outcome (10%) was: IPTW for ATE 0.1514 (rHRs = 1.1635); IPTW for ATT 0.0160 (rHRs = 1.0161); 3:1 PS matching 0.0758 (rHRs = 1.0787); PS Stratification 0.0177 (rHRs = 1.0179). A similar pattern for outcome prevalence was seen for all the simulation scenarios.

**Conclusion:**

This study showed that PS methods proposed in the literature may not all perform well for individual datasets. The findings produced recommendations for using PS methods in the estimation of real-world treatment effect when the covariate measurement error and sparse outcome data are present.

## Introduction

Although a Randomised Controlled Trial (RCT) is seen as the gold standard for estimating the effect of a novel treatment (Sibbald and Roland, 1998), the treatment effect in a real-world setting when it is prescribed to a more general population is likely to be different. Electronic Health Records (EHR) data offers the opportunity for the estimation of the real-world treatment effect from observational studies. However, the treatment allocation is not randomised so there are likely be systematic differences between the treatment groups, and, if this is not accounted for, the treatment effect estimate will be biased. Propensity Score (PS) methods (Rosenbaum and Rubin, 1983) are popular in applied medical research for adjusting for this treatment allocation bias.

PS analysis works within the Potential Outcomes Framework (or Counterfactual Framework) where every participant can have two potential outcomes. For participant *i* these are *Y*_*i*_(0) if the control treatment were received and *Y*_*i*_(1) if the novel treatment were received. The treatment effect for participant *i* would be *Y*_*i*_(1) − *Y*_*i*_(0). Each participant will only receive one of the treatments (the other is counterfactual) so this cannot be calculated. The observed outcome is *Y*_*i*_ = *Z*_*i*_ *Y*_*i*_(1) + (1 − *Z*_*i*_)*Y*_*i*_(0) where *Z* = 0 for the control treatment and *Z* = 1 for the novel treatment. Using all participants in the study population *E*[*Y*(1) − *Y*(0)] will give the Average Treatment Effect (ATE) (Imbens, 2004), that is the average effect of moving all those in the population from untreated to treated. The Average Treatment Effect of the Treated (ATT) is given by *E*[*Y*(1)– *Y*(0) |*Z* = 1 ] (Imbens, 2004), that is the average effect of moving those who actually received the active treatment from untreated to treated.

PS methods comprise of a range of approaches to balancing treatment groups, thus reducing the treatment allocation bias and hence obtaining a less biased estimate for the treatment effect estimate. The PS is the probability of receiving the novel treatment calculated from covariates thought to affect the treatment allocation and/or the outcome. This is regarded as a two-step approach; firstly the PS analysis is applied to adjust for treatment allocation bias and secondly the outcome analysis is performed. Propensity score methods provide a robust way to include the confounders (and competing treatments if necessary) in the outcome model and is rapidly becoming the ‘gold standard’ means to condition for confounders.

If the value of an observation does not match the true value of the quantity (or characteristic) being measured, this is known as ‘measurement error’ (Wallace, 2020). There has been a lack of application of measurement error methods within applied research (De Gil et al., 2015; Millimet, 2011). The types of measurement error which may occur in EHR are covariate measurement error, outcome measurement error and treatment allocation measurement error. The measurement error investigated in this study is non-differential measurement error, that is the measurement error is assumed to be the same for each treatment group, in a covariate in the treatment allocation model, the PS model. If covariate measurement error exists, the treatment groups will be balanced on the observed rather than the true covariates, so differences between the treatment groups will still exist, leading to a source of bias in the outcome analysis (Nguyen and Stuart, 2020). Its impact when using PS conditioning was demonstrated by Conover et al. (2021), De Gil et al. (2015) and Hong, Aaby, Siddique and Stuart (2019).

Measurement error adjustment methods which could be applied to similar data can be categorised as: generic methods, where the measurement error adjustment is applied to the data then the standard PS analysis is carried out; and specific to PS analysis, where the measurement error adjustment is combined with the PS analysis. Generic methods to address measurement error include: Multiple Over-imputation (MO) (Blackwell et al., 2017); Simulation Extrapolation (SIMEX) (Cook and Stefanski, 1994); Minimal Assumption Bounds (Black et al., 2000); Regression Calibration (Carroll and Stefanski, 1990). Methods which perform measurement adjustment specifically in PS analysis include: (Braun et al., 2017; Dong and Millimet, 2020; Hong et al., 2017; Raykov, 2012; Rudolph and Stuart, 2018; Webb-Vargas, Rudolph, Lenis, Murakami & Stuart, 2017). These methods were not used as the focus of this study is to investigate the effect of measurement error when using PS methods.

Another typical problem of EHR datasets is sparseness in data and it can be caused by any of the following: small sample size (Siino et al., 2018); rare exposure (treatment) (Hajage, Tubach, Steg, Bhatt & De Rycke, 2016); rare outcome events (Siino et al., 2018) which lead to a low number of events per variable (EPV) (Greenland et al., 2016); unbalanced or highly predictive risk factor variables (Siino et al., 2018) with narrow distributions or categories which are uncommon; variables which almost perfectly predict the outcome (Greenland et al., 2016); variables that together almost perfectly predict the exposure (treatment) (Greenland et al., 2016). Sparse outcome data are the focus of this study.

Sparse data bias produces treatment effect estimates away from the null, so inflated treatment effect estimates are produced. PS methods, Penalised Likelihood Estimation (PLE), Data Augmentation and Bayesian methods are some of the methods to avoid or reduce bias due to sparse data. The current study is limited to the use of PS methods. PS methods reduce sparse data bias by combining the information from several variables into one, making the number of EPV higher in the outcome model.

This study compares the performance of four commonly used PS methods when using EHRs to estimate the real-world treatment effect of a novel product in the presence of other real-world problems associated with EHR data: covariate measurement error and sparse outcome data. Generally the literature only considers one of these problems when comparing the performance of PS methods. The analysis was performed on an extract from The Health Improvement Network (THIN), UK primary care data, containing data from patients with atrial fibrillation (AF) prescribed the Novel Oral Anti-Coagulant (NOAC), Rivaroxaban, compared to the control, Warfarin an Oral Anti-Coagulant (OAC), in the prevention of future stroke. The outcome data were in time-to-event format with future stroke or TIA being an event of interest. The dataset represented typical real-world problems associated with EHR data: covariate measurement error and sparse outcome data.

## Methods

### Study dataset

This study used a data extract from THIN, one of the UK primary care datasets, supplied to the Performance-Based Innovation Rewards study (REWARD) (Banerjee et al., 2020), containing data for 21,259 patients with AF. The study dataset was selected using: the first NOAC/OAC prescription was for Rivaroxaban or Warfarin; the first NOAC/OAC prescription date was after the National Institute for Health and Care Excellence (NICE) approval date for the Rivaroxaban (May 2012); the patients were NOAC/OAC-naïve, meaning that this was the first NOAC/OAC prescription this patient was recorded as being prescribed. The analysis was to estimate the treatment effect of Rivaroxaban compared to Warfarin in the prevention of future stroke.

### Propensity score conditioning

The literature suggests the steps to run a PS analysis are: generate the PS model; check for common They implement measurement error in different ways is there is sufficient overlap of the PS distributions of the two treatment groups; apply the PS method; check for balance, that checks how well the PS model has been defined, and is implemented by checking that the PS method used has balanced the distribution of each covariate in the PS model between the treatment groups; estimate treatment effect (Austin, 2009a; Austin, 2011b; Garrido et al., 2014; Li, 2013). If any of the tests fail, then the PS model should be redefined and the process started again. This can be an iterative process.

Conditional on the true PS, treatment allocation is independent of the measured covariates. This means treated and untreated cases with the same true PS will have the same covariate distribution (Rosenbaum and Rubin, 1983). If the distribution of the covariates is similar for the cases with the same PS, then the PS is sufficiently well defined (Ho, Imai, King & Stuart, 2007). As the estimated PS is being generated, tests on the difference of the covariate distributions will indicate if the estimated PS is sufficiently close to the true PS (Austin, 2009a).

Logistic Regression was selected for the PS model in this study, as part of Generalized Linear Models family which are a traditional approach of directly adjusting for confounding. The literature presents different options for the selection of the covariates to include in the PS model. In this study, variables which influenced the prescribing (treatment allocation), were included in the PS model (although they may have affected the outcome).

Clinically relevant variables for the PS model were selected from advice by clinicians and supported by the literature: stroke (Hankey et al., 2012; Toso, 2014), alcohol misuse (Baczek et al., 2012), chronic kidney disease (Boriani et al., 2016), liver disease (Lai et al., 2016), CHA2DS2-VASc score the stroke risk score for patients with Atrial Fibrillation (Giralt-Steinhauer et al., 2013; Lee, Monz, Clemens, Brueckmann & Lip, 2012), HAS-BLED score the risk of major bleeding for patients on anticoagulation for AF (O'Caoimh et al., 2017), ischaemic heart disease used to indicate previous myocardial infarction (Bhatia and Lip, 2004), and age (Wolff et al., 2015). All clinically relevant variables were kept in the PS model, regardless of their statistical significance during the model selection process. Other non-clinically relevant variables which were seen to affect prescribing (identified from the data) were retained in the model if were statistically significant (p < 0.05). The ‘best’ model was selected, using the Bayesian Information Criterion (BIC) and further refined to give the PS model as shown in Table 1.

**TABLE 1:** The refined treatment allocation model for the RI-WA dataset.

Covariate	Coefficient	SE of coefficient	p	[95% CI]
Previous stroke	0.123	0.061	0.04	(0.004, 0.242)
Alcohol misuse	0.098	0.128	0.45	(-0.153, 0.348)
Chronic kidney disease	0.008	0.051	0.87	(-0.093, 0.109)
Liver disease	0.033	0.437	0.94	(-0.825, 0.890)
Ischemic heart disease	−0.082	0.051	0.11	(-0.181, 0.018)
First NOAC/OAC prescription was ≤28 days of first AF diagnosis?	−0.192	0.042	<0.001	(-0.275, −0.110)
= 86 if age≤86, else = age	0.077	0.013	<0.001	(0.053, 0.102)
Licence1*	0.153	0.011	<0.001	(0.131, 0.175)
Licence1^2^*	−0.001	<0.001	<0.001	(-0.002, −0.001)
Constant term	−10.830	1.096	<0.001	(-12.979, −8.682)

*Licence1 is the Rivaroxaban licence date to date of first NOAC/OAC, prescription, in months.

**FIGURE 1:**
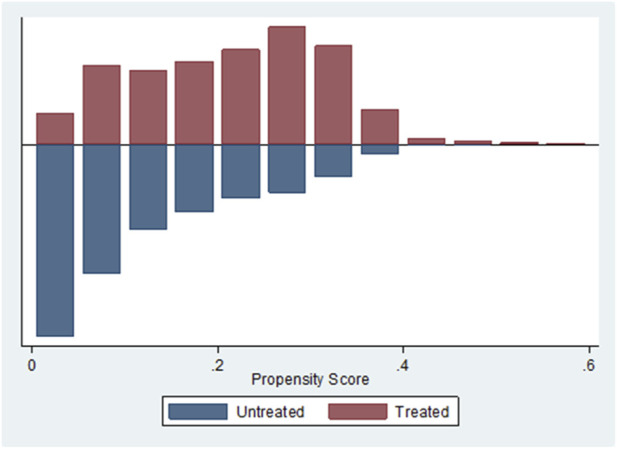
Histogram of Propensity Score, using Stata’s -psgraph-, for Rivaroxaban (Treated) and Warfarin (Untreated) for the RI-WA dataset.

Figure 1 shows that there was good common support in this dataset when using this model.

There are four general categories of methods for using PS to remove the effect of confounding, that is potential differences in characteristics between the treatment groups: PS matching, stratification on the PS, inverse probability treatment weighting (IPTW) on the PS and covariate adjustment on the PS (Austin, 2011a). In this study, PS matching, IPTW for ATE, IPTW for ATT and PS stratification were used. PS matching creates matched pairs, or groups, by matching each treated participant to one or more untreated participant with a similar PS. The estimate of treatment effect is generated from the matched sample or dataset, retaining only cases for whom a match is made, meaning that it is smaller than the full dataset used by other PS conditioning methods. Several PS matching methods were considered, (the details are presented in (Burnell, 2022) Appendix B-6.2, and 3:1 nearest neighbour matching with replacement was chosen for use as it generated a larger matched dataset with more outcome events than the 1:1 matching methods. The original dataset had considerably more Warfarin patients (18,348) than Rivaroxaban patients (2,911) which provided a pool of Warfarin controls for matching to the active Rivaroxaban cases. The Stata community-written command -psmatch2- (Leuven and Sianesi, 2003) was used to apply and implement this. The balancing checks which were applied to the matched dataset were: comparison of the PS distribution between the treatment groups, standardised differences of the variables and the number of matched pairs/groups generated, ((Burnell, 2022), Appendix B-6.3). These all confirmed that the PS matching had balanced the data sufficiently well.

IPTW on the PS (Rosenbaum, 1987) uses weights, based on the PS, to generate a synthetic dataset or sample. The weight is defined as the inverse probability of receiving the treatment the participant actually received. In this study, IPTW, implemented using the Stata community-written command -propwt- (Lunt & Linden, 2023), was used to estimate the ATE and ATT with a different formula used for the calculation of weights for the ATE and ATT. Balance checking was performed on a single run on the original dataset, with no measurement error added. The standardised means were compared between the treatment groups and the continuous variables plotted to compare their distributions between the treatment groups, (Burnell, 2022), Appendix B-6.4. These showed the weights applied for IPTW for ATT and IPTW for ATE balanced the standardised means of each variable in the PS model between the treatment groups.

Stratification on the PS (Rosenbaun and Rubin, 1984) is a form of subclassification, used to reduce bias. By stratifying on the PS, rather than the individual covariates, less strata are needed. The records of all participants are ordered by PS, then grouped into strata. The treatment effect is estimated within each stratum and then these stratum-specific results pooled, or similar, to generate the ATE and the SE of the estimate. Balance checking visually compared standardised differences of the variables between treatment groups following PS stratification with those in the original data, further details are provided in Supplementary Table S1 and also in (Burnell, 2022), Appendix B-6.5. PS stratification using 5, 10 and 50 strata all reduced the standardized differences. 10 strata were chosen for use in this study because it reduced the standardised differences more than 5 strata and each stratum was less sparse than when 50 strata were used.

### Outcome modelling

The primary outcome in the dataset was future stroke. The outcome analysis was performed on time-to-event data, that is time to first stroke following the first NOAC/OAC prescription, using survival analysis methods. Different implementations of Cox regression, which estimated treatment effect, were used to take account of the matched or weighted nature of the data. When using PS matching and PS stratification, Cox regression with stratification was used where each stratum was a matched pair or group in which the baseline hazard was assumed to be constant. When using IPTW, for both the ATE and ATT, the weights generated by IPTW were used directly as an option in the Cox regression. This weight was then used as Stata’s *pweight* (probability weights which represent the probability of the case being used in the sample and is proportional to the probability of the case being sampled) in the outcome analysis.

The outcome model was fitted to the analysis dataset used following PS matching. However, these same variables were used for the other PS methods, PS stratification, IPTW for ATE and IPTW for ATT, without refitting the model to the full dataset. This was done for consistency between the PS methods. The chosen model was the four clinically relevant variables identified as significant from the univariate modelling (prescribed blood pressure lowering medication, prescribed statins, prescribed antiplatelets and hypercholesterolemia), the CHA2DS2-VASc score (Lip et al, 2010) and treatment (Table 2). The estimated treatment effect is conditional on the variables in the outcome model. For use in the simulations a baseline hazard function was required. A Weibull distribution was fitted to the data because of the flexibility it offers, by varying its shape parameter, γ, the distribution of the function changes.

**TABLE 2:** The outcome model selected for use. The model includes treatment, the 4 most significant univariate variables and the CHA2DS2-VASc score.

Covariate	HR	SE of HR	95% CI of HR	Coeffi- cient*	SE of coeffi- cient	95% CI of coefficient	p- value
Treatment	1.534	0.383	(0.940, 2.504)	0.428	0.250	(-0.062, 0.918)	0.087
Prescribed blood pressure lowering medication	0.339	0.110	(0.180, 0.639)	−1.081	0.323	(-1.714, −0.448)	0.001
Prescribed statins	0.677	0.245	(0.333, 1.378)	−0.390	0.362	(-1.100, 0.321)	0.282
Prescribed antiplatelets	0.646	0.225	(0.326, 1.279)	−0.437	0.349	(-1.121, 0.246)	0.210
Hypercholesterolemia	0.729	0.269	(0.354, 1.502)	−0.316	0.369	(-1.039, 0.407)	0.391
CHA2DS2-VASc score	1.360	0.165	(1.073, 1.725)	0.308	0.121	(0.070, 0.545)	0.011

*Coefficient is the log(hazard ratio) - presented as it was used in the simulation process.

### Simulation method

The simulated datasets were generated using a *plasmode* simulation method (Vaughan et al., 2009) which is a resampling method where the draws of cases, that is ‘individual patient records’, are made from the original data and the resulting cases copied to the generated dataset. This preserves the relationship between the baseline variables for each case and hence accounts for and reflects the characteristics and specific features of the original real-world dataset. Once a dataset had been created, measurement error was introduced into the variable for previous stroke, to represent under- or over-recording of that variable, and an amended value generated for the PS value and CHA2DS2- VASc score. Variables for the simulated treatment allocation, simulated survival time and simulated survival outcome were created using the baseline variables, the chosen values for the effect size in the PS model of the variable with measurement error and the outcome prevalence. Under-recording was implemented as negative measurement error of previous stroke and over-recording of a was implemented as positive measurement error of previous stroke. The treatment effect was estimated from the dataset (using the simulated variables) and recorded. Performance measures of the PS methods, mean, SD, bias, mean squared error (MSE), percentage change MSE and mean Model SE, were calculated from the treatment effect estimates from all the generated datasets. The calculation for all simulation runs used an assumed true mean obtained from a single simulation run using the original dataset. It was generated as a plausible value to use in all simulations for estimations of both the ATE and the ATT. The implementation of the simulation experiment implies that the performance measures are relative to the assumed true mean estimated as 0.3674.

The number of simulations required for each PS method at each prevalence was chosen via a precision-based sample size calculation using an acceptable width of a 95%CI for the mean treatment effect estimate. The 95%CI was determined by calculating CI widths of the mean treatment effect estimate from simulations using 1,000 datasets, and determining an acceptable CI width. This was then used to calculate the number of simulations required. A rationale for choosing the acceptable CI width was based on the magnitude of the true value of the treatment effect, 0.3674, calculated using the dataset with the original characteristics. The CI width of no more than 10% of this effect parameter, 0.0367, was deemed as acceptable. This was supported by visual inspection of plots of the mean of the treatment effect estimate, from which a CI of 0.04 was thought to be too high and CI between 0.02 and 0.03 was thought to be acceptable. Combining this information, an acceptable CI width of 0.035 was decided upon. It is acknowledged that the selection of an acceptable CI is subjective.

**FIGURE 2:**
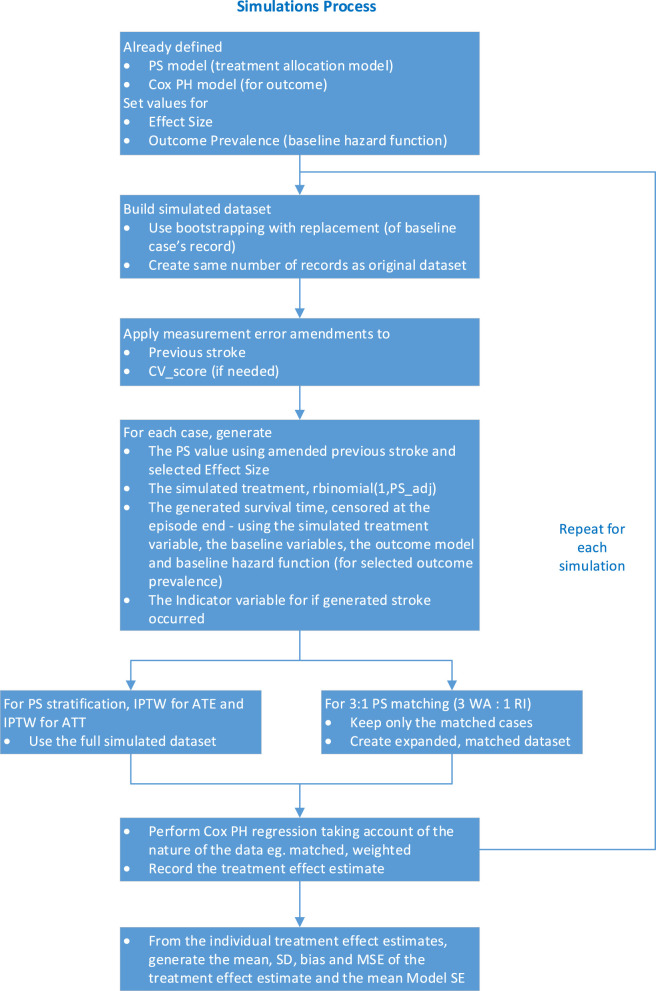
Flow diagram of the simulations process (CV_score is CHA2DS2-VASc score).

The simulation process is shown as a flow diagram in Figure 2. A dataset was generated using plasmode simulation, measurement error for previous stroke was introduced and additional variables generated using the previous stroke with measurement error and its effect size in the PS model. For the generated dataset, PS conditioning was applied and the outcome analysis performed using Cox PH regression and the treatment effect estimate recorded. This process was repeated for the required number of datasets and the performance measures calculated for the whole simulation run.

Primary care data may under-record events such as stroke, which are treated in secondary care, by 25%–35% (Burnell, 2015, Herrett et al., 2013). It is possible that there is over-recording of stroke so, to provide the complete picture, the current study looks at measurement error in both directions, that is both under-recording and over-recording. The chosen range of the measurement error was expanded to - 50% to +50%, which included the provisional estimate of 25%–35% under-recording.

Coefficients to represent high, medium and low effect size for previous stroke were generated using the following method.

The Odds Ratio (OR) of interest is

$$OR=\frac{odds\;\;of\;\;receiving\;\;Rivaroxaban\;\;if\;had\;\;previous\;\;stroke}{odds\;\;of\;\;receiving\;\;Rivaroxaban\;\;if\;no\;\;previous\;\;stroke}$$

If $\beta_{1}$ is the coefficient for previous stroke in the PS model, then $OR=\exp\left(\beta_{1}\;\right)$ Cohen’s *d* is the standardised mean difference between two group means, the effect size underlying power calculations for the two-sample t-test (Cohen, 1988). Cohen’s *d* = 0.2, 0.5, and 0.8 are often used to indicate a low, medium, and high effect size (Chen, Cohen and Chen, 2010). Chen, Cohen and Chen (2010) calculated the Odds Ratios (OR) equivalent to Cohen’s *d*, for low, medium and large effect size and presented the OR for different disease rates in the non-exposed group.

The calculated values of ORs, which were relevant to this study, are given in Table 3 and informed the values of the coefficient used to represent the low, medium and large effect size based on Cohen’s *d*. The coefficient of previous stroke in the PS model was generated as $\ln\left(OR\right)$ . This value was supplied as a parameter to the simulations and used in the PS model.

**TABLE 3:** Effect sizes for prevalence of RI is generated treatment of 1% and 10%.

Prevalence**	Low effect Cohen’s d = 0.2			Medium effect Cohen’s d = 0.5			Large effect Cohen’s d = 0.8		
	OR	Coefficient	Xorig*	OR	Coefficient	Xorig*	OR	Coefficient	Xorig*
0.01 (1%)	1.6814	0.519627	4.2	3.4739	1.24528	10.1	6.7128	1.90402	15.5
0.1 (10%)	1.4615	0.379463	3.1	2.4972	0.91517	7.4	4.1387	1.42038	11.6

* The multiple of the original coefficient, 0.1229108.

**Prevalence of RI (novel treatment) is generated treatment.

This change of effect size related to the PS modelling which was used to correct for treatment allocation bias. The ‘outcome’ in this case was the generated treatment, which was created using the participant’s PS value. The ‘untreated’ group was those with no previous stroke, those with a generated treatment of Rivaroxaban (the NOAC) contributed to the ‘outcome prevalence’. In the study data the outcome prevalence took values between 13% and 14.2%, so the values quoted for the 10% prevalence in Table 3 were used to change the effect size of previous stroke in the PS model. Rounding these parameters for use in the simulation runs, the coefficient of previous stroke in the PS model took the values of 0.5 for low effect size, 1.0 for medium effect size and 1.5 for large effect size. When the PS model was fitted to the original data, the effect size of previous stroke was 0.123. This was ‘very low’ compared with Cohen’s classification. Simulations using this very low (or original) effect size are also presented.

For the outcome prevalence the values chosen for the simulation experiment were approximately 1% prevalence, which is similar to the original dataset, approximately 0.5% prevalence to investigate the effect of a lower prevalence, and approximately 10% prevalence to investigate the effect of data which does not suffer from substantial sparseness of outcomes. This was implemented in the Weibull baseline hazard function by varying λ (the scale parameter) and keeping γ (the shape parameter) constant.

In summary the parameters used in the simulations are given in Table 4.

**TABLE 4:** Parameters and their values used in the simulation runs.

Parameter	Values
Measurement error in previous stroke in the PS model	−50%, −30%, −10%, 0%, +10%, +30%, +50%
Effect size of variable with measurement error	0.123 (original), 0.5 (low), 1.0 (medium) and 1.5 (high)
Outcome prevalence of future stroke	0.5%, 1%*, 10%
Sample size, N, the number of simulated datasets	Specific to each PS method for each outcome prevalence

*Outcome prevalence in the original study dataset.

A separate heatplot is displayed for each performance measure, using the same layout. On the y-axis, each PS method reports the results from the effect sizes of the covariate in the PS model with measurement error, Original, Low, Medium and High. On the x-axis within each outcome prevalence, 0.5%, 1% and 10%, results are reported for the measurement error, −50% to +50%. Each cell therefore reports the value of the performance measure for the given PS method, effect size of the covariate in the PS model with measurement error, outcome prevalence and measurement error of the covariate in the PS model.

## Results

**FIGURE 3:**
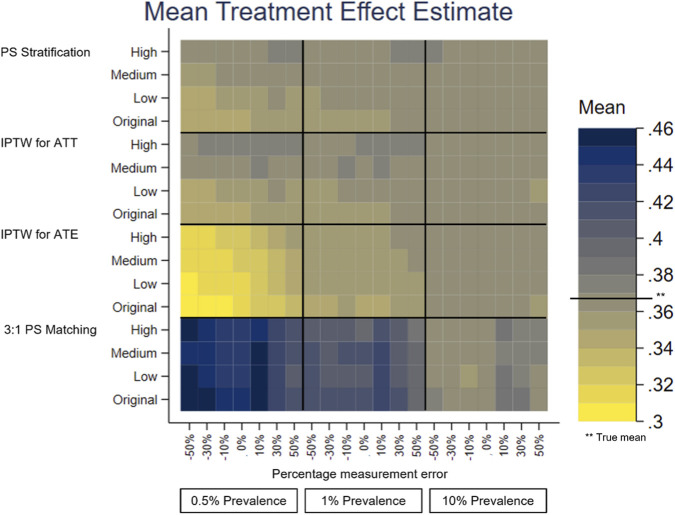
Heat plot for Mean treatment effect estimate. The x-axis shows the outcome prevalence and the measurement error. The y-axis shows the PS method and the ‘effect size’ used. The horizontal line in the key indicates the true mean.

**FIGURE 4:**
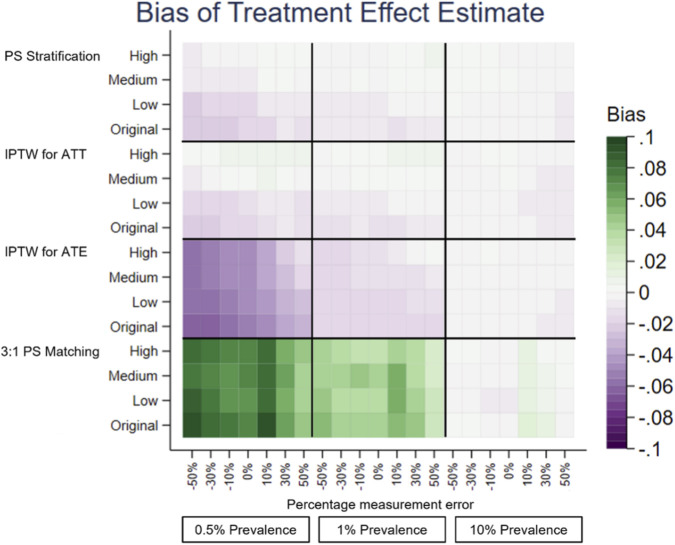
Heat plot for the Bias of the treatment effect estimate. The x-axis shows the outcome prevalence and the introduced measurement error. The y-axis shows the PS method and the ‘effect size’.

**FIGURE 5:**
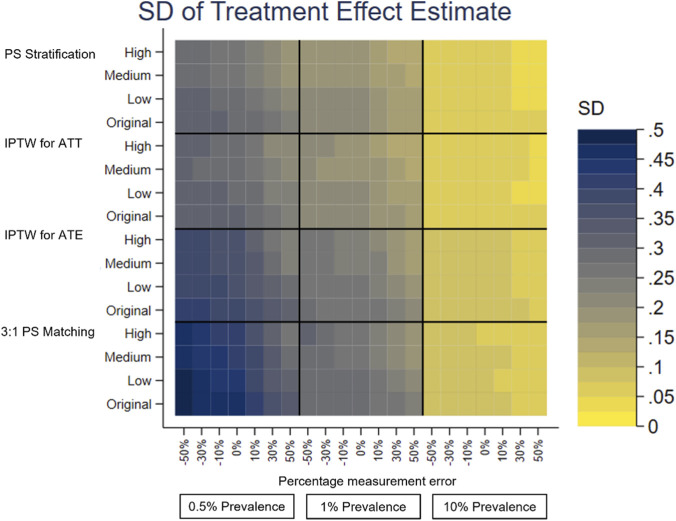
Heat plot of the SD of the treatment effect estimate. The x-axis shows the outcome prevalence and the introduced measurement error. The y-axis shows the PS method and the ‘effect size’ used.

**FIGURE 6:**
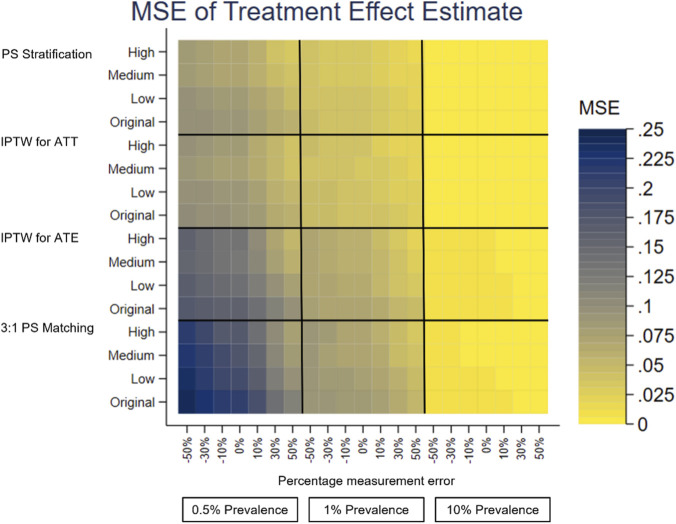
Heat plot of the MSE of the treatment effect estimate. The x-axis shows the outcome prevalence and the introduced measurement error. The y-axis shows the PS method and the ‘effect size’ used.

**FIGURE 7:**
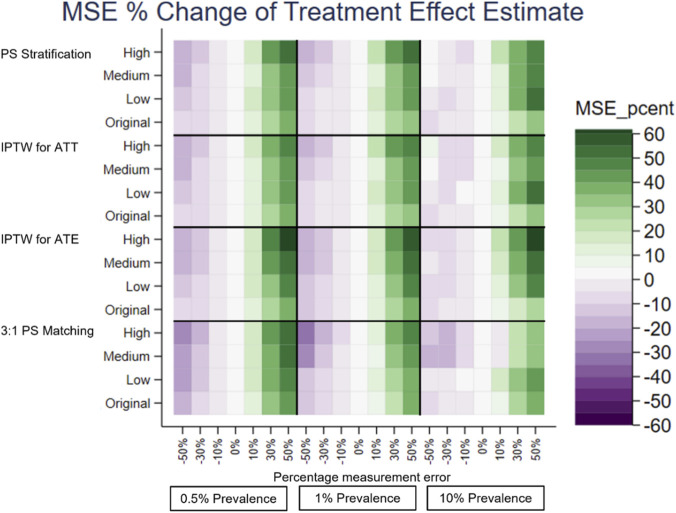
Heat plot of the MSE percentage change of the treatment effect estimate. The x-axis shows the outcome prevalence and the introduced measurement error. The y-axis shows the PS method and the ‘effect size’ used.

**FIGURE 8:**
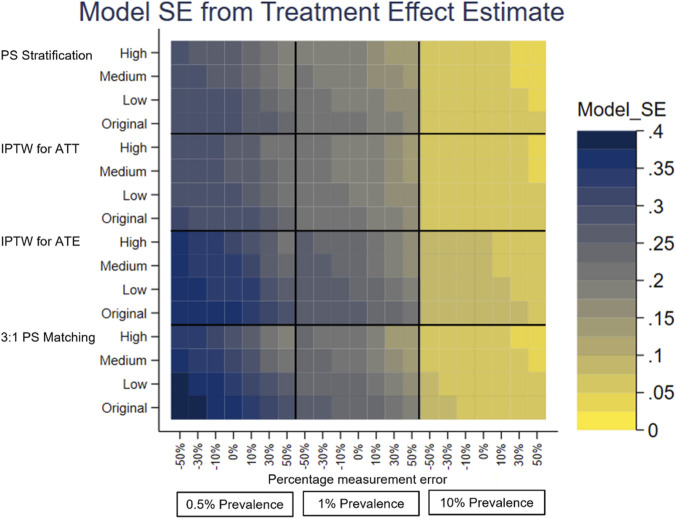
Heat plot of the Model SE of the treatment effect estimate. The x-axis shows the outcome prevalence and the introduced measurement error. The y-axis shows the PS method and the ‘effect size’ used.

The performance measures of the treatment effect estimate generated presented as mean, SD, bias, absolute MSE, percentage change MSE and model SE are displayed in the heat plots, Figures 3–8. The results display the estimate of the treatment effect (of Rivaroxaban over Warfarin) presented as the log(HR).

Comparing all four PS methods using simulations was firstly based on the original characteristics of the data–no measurement error, the original effect size of previous stroke in the treatment allocation model and an outcome prevalence of 1%. 3:1 PS matching (using nearest neighbour matching with replacement) appeared to perform the least well of the PS methods. It had larger bias and the bias was positive, 0.0428, as opposed to negative for the other PS methods, IPTW for ATE (−0.0181), IPTW for ATT (−0.0110) and PS stratification (−0.0099).

For illustration and to aid clinical interpretability of the results, the bias figures above, based on the original characteristics of the data, were transformed to a HR scale. The biases would correspond to the following rHRs (ratio of HR for given method to the “true” HR = 1.4440 corresponding to the true value of the treatment effect 0.3674, HRs of future stroke with Warfarin vs. Rivaroxaban): 1.0437 for 3:1 PS matching, 0.9821 for IPTW for ATE, 0.9891 for IPTW for ATT and 0.9901 for PS stratification. These would correspond to the following HRs accordingly: 1.5071, 1.4181, 1.4282 and 1.4298, with differences between numbers being small to negligible. 3:1 PS matching was retained for use in the later simulations to assess its performance with varying measurement error, effect size and outcome prevalence.

Under-recording of a variable in the treatment allocation model was implemented as negative measurement error of previous stroke [-50%, 0%). Previous stroke had a very small effect on treatment allocation. All PS methods used in this study showed there was little change in the bias of the treatment effect estimate and there was slightly lower precision and a small increase in the MSE for increasing the magnitude of negative measurement error. The increase in the MSE from 0% to −50% measurement error in the treatment allocation model was, IPTW for ATE 0.0075, IPTW for ATT 0.0025, 3:1 PS matching 0.0088 and PS Stratification 0.0028.

Over-recording of a variable in the treatment allocation model was implemented as positive measurement error of previous stroke (0%, +50%]. In common with under-recording all PS methods used in this study showed there was little change in the bias of the treatment effect estimate. Over the measurement error range, as the size of the over-recording increased, the precision in the estimation increased as did the MSE. The increase in the MSE from +50% to 0% measurement error in the treatment allocation model was, IPTW for ATE 0.0261, IPTW for ATT 0.0149, 3:1 PS matching 0.0297, PS Stratification 0.0145. Higher over-recording of previous stroke generates a higher prevalence of stroke, generating more outcomes of future stroke, which in turn increases the precision of the treatment effect estimate, hence a lower MSE.

The impact that the variable with under- or over-recording has on determining the treatment allocation (the effect size) was varied using values representing Low, Medium and High for comparison with the Original (very low) effect size. There was still little variation in the mean, and bias, over the measurement error range for all the effect sizes. When the variable with measurement error had greater impact on the treatment allocation model, (the PS model), the treatment effect estimate had lower bias and higher precision. For example, using the characteristics of the original dataset the reduction in bias from small effect size to high effect size is IPTW for ATE 0.0029, IPTW for ATT 0.0037, 3:1 PS matching 0.0062, PS Stratification 0.0041. It does seem counterintuitive and could be due to the Data Generation Mechanism (DGM) used. In particular, when the variable with measurement error (previous stroke) had a high effect size in the PS model, for those with a previous stroke, their PS value will be higher than if the effect size were low. A higher PS value increases the probability of the generated treatment being Rivaroxaban. This in turn generates more outcome events in the simulations, making the outcome modelling more stable and the treatment effect estimate having lower bias and higher precision.

To investigate the impact of sparse outcome data, the outcome prevalence was varied by generating data with different numbers of participants with future strokes (the primary outcome). The lower prevalence (≤1%) data gave treatment effect estimates with a higher bias and lower precision and using the higher prevalence data, with lower bias and higher precision. These results were to be expected, as higher EPV in the outcome model generates more stable models. For example, with no added measurement error, the difference in the bias of the low prevalence data (0.5%) and the high prevalence data (10%) is IPTW for ATE 0.1514, IPTW for ATT 0.0160, 3:1 PS matching 0.0758, PS Stratification 0.0177. At lower prevalences, there was more variation in the performance measures of the treatment effect estimate over the measurement error range of a variable in the treatment allocation model.

The differences in the performance measures of the treatment effect due to different effect sizes are greater when the data has lower outcome prevalence. The treatment effect estimates with the highest bias, lowest precision and highest MSE were obtained with low prevalence outcome data and when the variable with measurement error had a low (or very low) impact in the PS model.

PS stratification and IPTW for ATE were the PS methods used which estimate the ATE. Both methods gave treatment effect estimates which followed the patterns described above in all the simulation scenarios. There was only a little difference in the bias from both methods, but the bias was slightly closer to 0 for PS stratification. PS stratification had a higher precision and lower MSE, than those for IPTW for ATE over the measurement error range.

3:1 PS Matching and IPTW for ATT were the PS methods used which estimate the ATT. Both methods gave treatment effect estimates which followed the patterns described above in all the simulation scenarios. In all scenarios IPTW for ATT had lower bias and higher precision than 3:1 PS Matching.

## Discussion

The real-world treatment effect can be estimated from EHR by forming an observational study, in which the treatment allocation is not randomised and PS methods are used to adjust for treatment allocation bias, prior to fitting a statistical model to the outcome data. The aims of this study were to investigate the effect of under- or over-recording of a dichotomous covariate in the treatment allocation model with sparse outcome data when estimating the real-world treatment effect via PS methods, and to compare the performance of different PS methods in these scenarios. The simulation experiments were rigorously designed and implemented to account for and reflect the characteristics and specific features of the real-world dataset used.

When using PS methods to correct for treatment allocation bias, the bias of the treatment effect estimate appears to be robust to measurement error/misclassification of a variable with very low impact in the treatment allocation model. When there is more under-recording of this variable, there is lower precision in this estimate. When there is more over-recording of this variable, the method produces an estimate with higher precision. Under-recording or over-recording in a covariate which affects the treatment allocation does not affect the relative performance of the PS methods used in this study. Although there are a small number of studies which report on the effect of varying measurement error and compare PS methods their study designs are different to the current one. They implement measurement error in different ways and use different performance measures, making a direct comparison with the current study difficult. In De Gil et al. (2015) covariate measurement error affected bias but not the root mean squared error (RMSE), which is different to the current study. Conover et al. (2021) reported that scenarios with only false positive misclassifications (over-recording) produced higher bias than scenarios with only false negative misclassifications (under-recording). In the current study there was little variation in the bias and any change was in the opposite direction, with slightly lower bias for over-recording (positive measurement error). Hong et al. (2019) showed that the bias and MSE reduced as the reliability of mismeasured confounders approached one (i.e. as measurement error approached zero). These results are different to the current study, as positive measurement error behaved in a different manner than negative measurement error. This could be due to the small changes to the number of outcomes that the measurement error produced, which is noticeable when the outcome prevalence is low, close to 1%. Varying the effect size of the covariate in the treatment allocation model with measurement error made little difference to the simulation results. The simulations in this study were run to investigate the effect of under-recording or over-recording of a single covariate. Also, there is likely to be measurement error in several covariates and the primary outcome variable (future stroke). These were not considered in this study and further research is required to explore effect of errors in multiple co-variates.

The study dataset had rare (sparse) outcomes (prevalence approx. 1%) which could lead to a low EPV in the outcome models, hence bias in the outcome modelling. This type of sparse data, a large dataset with rare outcomes, is not uncommon (Chao, 1994; Franklin et al., 2017; Paul and Deng, 2000). Even though the outcome may be rare, it can be serious such as Serious Adverse Events in drug studies (Ross et al., 2015) and neonatal trials (Das et al., 2016). Few papers compare PS methods in the presence of sparse data, the exceptions being Franklin et al. (2017) for rare outcomes; and Hajage et al. (2016) for a rare exposure.

When varying the degree of under- or over-recording of a dichotomous variable in the treatment allocation model in addition to varying the outcome prevalence, the lower prevalence (≤1%) data gave treatment effect estimates with a higher bias and lower precision, whereas using the higher prevalence data gave a treatment effect estimate with lower bias and higher precision. The difference in the bias of the low prevalence outcome (0.5%) and the high prevalence outcome (10%) is: IPTW for ATE 0.1514; IPTW for ATT 0.0160; 3:1 PS matching 0.0758; PS Stratification 0.0177. At lower prevalences, there was more variation in the performance measures of the treatment effect estimate over the measurement error range of a variable in the treatment allocation model. Studies using EHR with lower prevalence outcomes should take account of covariate measurement error.

The recommendation for the PS methods to use remained the same in all simulation scenarios (no introduced measurement error, introduced measurement error, introduced measurement error and varied effect size, introduced measurement error and varied outcome prevalence and introduced measurement error, varied effect size and varied outcome prevalence). Studies which compare the performance of PS methods seldom include both the effect of covariate measurement error and sparse outcome data, nor do they use time-to-event data. PS matching and IPTW are generally recommended for binary outcome data (Austin, 2009b; Austin, 2011a; Austin, 2011b) and also for time-to event outcomes (Austin, 2013). However, the properties of the dataset may guide the choice of PS conditioning (Busso et al., 2014).

Based on this study’s data, for estimation of the ATE, PS stratification performed slightly better than IPTW for ATE. Its bias was similar to IPTW for ATE but its precision was higher (with a lower MSE), but the difference in performance was small. In the literature there were some reservations about the balance produced by PS stratification. The literature generally recommends IPTW for ATE as it provides the best balance (Austin, 2009b). However, Franklin et al. (2017)’s averaged results from simulations varying outcome prevalence recommended PS stratification (using 10 strata) over IPTW for ATE in terms of absolute bias and MSE.

Based on this study’s data, the recommendation was to use IPTW for ATT for estimating the ATT, which showed superior performance over 3:1 PS matching using nearest neighbour matching with replacement. In all scenarios IPTW for ATT had lower bias and higher precision. PS matching and IPTW are reported to remove systematic differences to a similar extent (Austin, 2009b; Austin, 2011a; Austin, 2011b) and in some cases, PS matching is recommended over IPTW for ATT (Conover et al., 2021). Hajage et al. (2016) found IPTW for ATT performed better than PS matching using time-to-event data with sparseness in the exposure data (rare treatment).

This study has shown that PS methods recommended in the literature may not perform well for individual datasets and specific scenarios such as for time-to-event data. Although Caliendo and Kopeinig (2008) and Garrido et al. (2014) recommended applying several PS methods and selecting the one which produces the best balance for the outcome analysis, this study showed that PS methods which give relatively poor balance can produce a treatment effect estimate with lower bias and higher precision.

While most studies use binary outcomes, potentially time-to-event data might be used when there are few events (as it gives the small improvement in power), though it is also commonplace to consider these events simply as binary outcomes. For data characterised by sparse outcomes, the influence of both measurement error and effect size is amplified; consequently, their impact may be more pronounced in time-to-event analyses. When the outcome data are ‘time-to-event’ data, guidance on the implementation of PS methods, particularly PS matching, and comparison of PS methods should be considered for future work. Further exploration of the modelling options to account more rigorously for the matched nature following PS matching of the data when Cox regression is performed could be undertaken.

The variables in the outcome model were selected by fitting the model to the analysis dataset used following PS matching. The same variables were used for the other PS methods without refitting the model to the full dataset. This may be regarded as a limitation of this study that the outcome model may be regarded as misspecified. Another limitation of this work is that in the estimation of treatment effect variance no account was taken of the uncertainty in estimating the PS. This means the CIs of the treatment effect produced were too wide. For PS stratification, using the analogous marginal variance method, which accounts for the uncertainty from estimating the PS model from the data, can reduce the variance by up to 12% depending on the data characteristics, compared to the commonly used variance estimation (Williamson et al., 2012). For IPTW the variance reduction can be 18% using the analogous marginal variance method compared to the commonly used variance estimation. Such changes are particularly noticeable for larger samples, n > 1000 (Williamson et al., 2012). Use of the analogous marginal variance method may affect the performance of the different PS methods compared in this paper and requires further investigation.

This study used four PS methods (each with one outcome option) which is a limitation, as there are many variations of these basic categories of PS methods. It would be reasonable to assume that other PS methods may behave in a similar way, that is for lower outcome prevalences, more variation in the performance measures of the treatment effect estimate over the measurement error range of a variable in the treatment allocation model. To identify the most effective PS methods for estimating the ATT and ATE, a thorough comparative simulation is required similar to that conducted in this study. Also, it is currently not clear if the changes to the treatment effect estimate are due to changes in the effect size or the DGM used. The parameters varied in the simulations only took a limited range of values (see above for suggested extensions of the simulations). The results using additional PS methods or expanded parameter ranges could be compared with those of this study.

Both the US Food and Drug Administration draft guidance on real-world evidence (RWE) and Guidance on Non-Interventional Studies (ICH M14) of the European Medicines Agency incorporate principles of causal inference and recommend using rigorous study design and analytical methods, such as those within the causal inference framework, to minimise bias and generate reliable evidence from observational data. They emphasise that studies using RWE often aim to evaluate a causal association and that approaches to address bias and confounding are critical. The results of this study provide guidance for researchers in selecting appropriate PS methods and designing effective and robust analysis strategies to mitigate bias when inferring causality from observational data.

## Summary/conclusion

Studies which use EHR to conduct observational studies should consider the impact of sparse outcome data, not just on the bias and precision of the treatment effect estimate, but also on the effect that covariate measurement error on the treatment allocation will have. For data with outcomes in the form of time-to-event, not all outcome events will be recorded in the dataset as some will be censored, making the data more sparse, which compounds these problems.

The findings of this study contribute to the body of knowledge, particularly when using PS methods and varying covariate measurement error, the covariate’s effect size in the treatment allocation model and the outcome prevalence. Systematically varying a combination of these parameters constitutes novel approach. These simulations were applied to time-to-event data, which are generally not widely reported on. The majority of studies which compare PS methods, investigate the effect of measurement error or sparseness of data use data with binary outcomes. This study did make recommendations for the PS methods to use, but the recommendations have been guided by the characteristics of the study dataset, which may mean that they are limited to datasets with similar characteristics rather than being more widely generalisable.

## Data Availability

The data analyzed in this study is subject to the following licenses/restrictions: The data were held by the University of Birmingham, approval for access to the data was granted for this study. Requests to access these datasets should be directed to ST, stishkovskaya@lancashire.ac.uk, for clarification of further enquires.

## Glossary

AF: Atrial Fibrillation
ATE: Average Treatment Effect
ATT: Average Treatment Effect on the Treated
BIC: Bayesian Information Criteria
CHA2DS2-VASc: Stroke risk score for patients with Atrial Fibrillation
CI: Confidence Interval
DGM: Data Generation Mechanism
EHR: Electronic Health Record
EPV: Events Per Variable
HAS-BLED: Risk score for bleeding in patients with Atrial Fibrillation
HR: Hazard ratio
IPTW: Inverse Probability of Treatment Weighting
MO: Multiple Overimputation
MSE: Mean Squared Error
NICE: National Institute for Health and Care Excellence
NOAC: Novel Oral Anti-Coagulant
OAC: Oral Anti-Coagulant
OR: Odds Ratio
PH: Proportional Hazards
PLE: Penalized Maximum Likelihood
PS: Propensity Score
RCT: Randomised Control Trial
rHR: Relative hazard ratio/ratio of hazard ratios
RI: Rivaroxaban
RMSE: Root Mean Squared Error
SD: Standard Deviation
SE: Standard Error
SIMEX: Simulation Extrapolation
THIN: The Health Improvement Network
WA: Warfarin
