# Supplementary material for: Estimating real-world treatment effects in the presence of measurement error and sparse outcome data using propensity score methods
Source: Front Pharmacol. 2026 Mar 9;17:1380586. doi: 10.3389/fphar.2026.1380586 (PMC13006843; doi:10.3389/fphar.2026.1380586)
Supplement: Supplementary file 1 [file Table1.docx]

**Supplementary material**

Balance checking for PS Stratification

The results of the balance checking following PS stratification are given in Table S1.

*Table S1: Standardised mean differences for the original data and that stratified on the PS with 5, 10 and 50 strata.*

| Number of Strata | Covariate | Mean in treated | Mean in untreated | Standardised diff. |
| --- | --- | --- | --- | --- |
| Original Data | Previous stroke | 0.14 | 0.14 | 0.010 |
|  | Alcohol misuse | 0.03 | 0.03 | 0.019 |
|  | Chronic kidney disease | 0.22 | 0.22 | 0.014 |
|  | Liver disease | 0 | 0 | 0.001 |
|  | Ischemic heart disease | 0.22 | 0.24 | -0.043 |
|  | af_to_noac_gen† | 0.44 | 0.47 | -0.060 |
|  | =86 if age≤86, else =age | 0.58 | 0.37 | 0.130 |
|  | licence_to_noac30†† | 24.32 | 15.79 | 0.934 |
|  | (licence_to_noac30)^2^ | 660.82 | 346.86 | 0.874 |
|  |  |  |  |  |
| Number of Strata | Covariate | Mean in treated | Mean in untreated | Standardised diff. |
| 5 Strata | Previous stroke | 0.14 | 0.14 | 0.009 |
|  | Alcohol misuse | 0.03 | 0.03 | 0.004 |
|  | Chronic kidney disease | 0.22 | 0.22 | 0.008 |
|  | Liver disease | 0 | 0 | 0.003 |
|  | Ischemic heart disease | 0.22 | 0.22 | -0.006 |
|  | af_to_noac_gen† | 0.44 | 0.45 | -0.019 |
|  | =86 if age≤86, else =age | 0.58 | 0.51 | 0.043 |
|  | licence_to_noac30†† | 24.32 | 23.90 | 0.046 |
|  | (licence_to_noac30)^2^ | 660.82 | 641.67 | 0.053 |
|  |  |  |  |  |
| Number of Strata | Covariate | Mean in treated | Mean in untreated | Standardised diff. |
| 10 Strata | Previous stroke | 0.14 | 0.14 | 0.004 |
|  | Alcohol misuse | 0.03 | 0.03 | -0.001 |
|  | Chronic kidney disease | 0.22 | 0.22 | 0.004 |
|  | Liver disease | 0 | 0 | 0 |
|  | Ischemic heart disease | 0.22 | 0.22 | -0.002 |
|  | af_to_noac_gen† | 0.44 | 0.44 | -0.006 |
|  | =86 if age≤86, else =age | 0.58 | 0.53 | 0.029 |
|  | licence_to_noac30†† | 24.32 | 24.24 | 0.010 |
|  | (licence_to_noac30)^2^ | 660.82 | 656.18 | 0.013 |
|  |  |  |  |  |
| Number of Strata | Covariate | Mean in treated | Mean in untreated | Standardised diff. |
| 50 Strata | Previous stroke | 0.14 | 0.14 | -0.001 |
|  | Alcohol misuse | 0.03 | 0.03 | -0.004 |
|  | Chronic kidney disease | 0.22 | 0.22 | 0.002 |
|  | Liver disease | 0 | 0 | -0.002 |
|  | Ischemic heart disease | 0.22 | 0.22 | 0.001 |
|  | af_to_noac_gen† | 0.44 | 0.44 | 0 |
|  | =86 if age≤86, else =age | 0.58 | 0.56 | 0.010 |
|  | licence_to_noac30†† | 24.32 | 24.30 | 0.002 |
|  | (licence_to_noac30)^2^ | 660.82 | 659.25 | 0.004 |

†First NOAC/OAC prescription was ≤ 28 days of first AF diagnosis?

††The Rivaroxaban licence date to date of first NOAC/OAC prescription, in months
